# The transmission line foreign body detection algorithm based on weighted spatial attention

**DOI:** 10.3389/fnbot.2024.1424158

**Published:** 2024-06-26

**Authors:** Yuanyuan Wang, Haiyang Tian, Tongtong Yin, Zhaoyu Song, Abdullahi Suleiman Hauwa, Haiyan Zhang, Shangbing Gao, Liguo Zhou

**Affiliations:** ^1^School of Computer and Software Engineering, Huaiyin Institute of Technology, Huaian, Jiangsu, China; ^2^Institute of Eco-Chongming (IEC), Shanghai, China

**Keywords:** transmission lines, WSA, LSKNet, BiFPN, BSAM

## Abstract

**Introduction:**

The secure operation of electric power transmission lines is essential for the economy and society. However, external factors such as plastic film and kites can cause damage to the lines, potentially leading to power outages. Traditional detection methods are inefficient, and the accuracy of automated systems is limited in complex background environments.

**Methods:**

This paper introduces a Weighted Spatial Attention (WSA) network model to address the low accuracy in identifying extraneous materials within electrical transmission infrastructure due to background texture occlusion. Initially, in the model preprocessing stage, color space conversion, image enhancement, and improved Large Selective Kernel Network (LSKNet) technology are utilized to enhance the model's proficiency in detecting foreign objects in intricate surroundings. Subsequently, in the feature extraction stage, the model adopts the dynamic sparse BiLevel Spatial Attention Module (BSAM) structure proposed in this paper to accurately capture and identify the characteristic information of foreign objects in power lines. In the feature pyramid stage, by replacing the feature pyramid network structure and allocating reasonable weights to the Bidirectional Feature Pyramid Network (BiFPN), the feature fusion results are optimized, ensuring that the semantic information of foreign objects in the power line output by the network is effectively identified and processed.

**Results:**

The experimental outcomes reveal that the test recognition accuracy of the proposed WSA model on the PL (power line) dataset has improved by three percentage points compared to that of the YOLOv8 model, reaching 97.6%. This enhancement demonstrates the WSA model's superior capability in detecting foreign objects on power lines, even in complex environmental backgrounds.

**Discussion:**

The integration of advanced image preprocessing techniques, the dynamic sparse BSAM structure, and the BiFPN has proven effective in improving detection accuracy and has the potential to transform the approach to monitoring and maintaining power transmission infrastructure.

## 1 Introduction

The integrity and reliability of power transmission systems are paramount for maintaining a stable electricity supply, which is fundamental to modern society and economic activities. Foreign objects on transmission lines, such as tree branches, plastic bags, or other debris, pose a significant threat to this integrity. These objects can cause short circuits, power outages, and even catastrophic failures leading to substantial economic losses and potential safety hazards. The presence of foreign objects can also lead to line tripping, which disrupts the power supply and affects the quality of electricity delivery to consumers. Presently, manual inspection is the main method of inspection for most transmission lines (Koh, 2023; Luo et al., 2023). However, transmission lines often exist in complex natural environments and harsh weather conditions, such as mountains, forests, and deserts. Harsh environments and weather conditions cause manual inspection problems, which lead to false alarms, missed reports and low detection rates. These constraints hinder the prompt detection of foreign objects on power lines, challenging the timely identification and resolution of potential safety risks. Technological limitations, environmental factors, and the need for improved data processing and response systems are key areas that require attention to enhance real-time monitoring and safety on power lines. In addition, manual inspections are costly, and it is difficult to cover all transmission lines. These problems have led to new requirements for ensuring the operation and the upkeep of high-voltage electrical networks, making it difficult for conventional manual monitoring techniques to meet actual needs in complex environments and real-time monitoring of foreign objects on numerous transmission lines.

In this context, improving the efficiency and accuracy of transmission line inspections has become an urgent problem. Many researchers are committed to developing intelligent inspection systems for power line monitoring utilizing deep learning to ensure effective and precise assessments.

Wang et al. (2022) proposed a fusion detection model based on multiscale appearance and relationship features. In comparison to the initial YOLOv5, the suggested model demonstrated enhanced precision. However, this model's performance is still limited when dealing with images that have highly similar background textures, possibly due to a lack of in-depth understanding of local and global contexts. Wang et al. (2023a) proposed a fusion detection model based on the improved YOLOv8m. In a foreign transmission line object detection model, the model's architecture was optimized by substituting the original SPPF component with an advanced SPPCSPC component, thereby bolstering its capacity for extracting multi-scale features. Additionally, the implementation of the Focal-EIoU loss function addressed the issue of sample quality imbalance, ensuring a more equitable focus on both high-quality and low-quality samples during training. However, this method may over-rely on the re-weighting of samples and does not fully consider the dynamism of feature extraction. Liang et al. (2020) investigated a deep neural network approach for assessing and pinpointing flaws in power line inspections, employing learned transfer and parameter optimization techniques to build the Fast R-CNN detection model. Nevertheless, the model's performance may decline when dealing with small or partially occluded targets, indicating the need for more refined feature extraction and target localization mechanisms. Wang et al. (2017) proposed a YOLOv5 transmission line inspection image detection model implementing the K-means clustering technique to refine the dimensions of the anchor boxes, this method enhances the model's capability to accurately align with salient object characteristics. The generalizability of this method may be limited under different environmental conditions, especially in complex and variable outdoor settings. Liang et al. (2023) proposed optimizing the YOLOv5s model to solve the problems of low accuracy and poor timeliness of deep learning network models in processing background texture occlusion images. The threshold function is used to denoise the image, and the original loss function GIOU_Loss is optimized into the CIOU_Loss function, which is subsequently fine-tuned. While these improvements have increased the model's robustness to some extent, adaptability to dynamic environmental changes remains a challenge.

Although many scholars have achieved excellent results within the context of foreign object detection on power lines, the accuracy of current technology is still insufficient for increasingly complex transmission line networks. For example, in complex environments, due to background occlusion and light, foreign objects in power transmission lines cannot be completely identified in the picture, causing foreign objects to be occasionally missed. In response to the limitations of existing models, the WSA model proposed in this paper introduces a weighted spatial attention mechanism to enhance the capture of local features and the understanding of global contexts. By dynamically adjusting attention weights, the WSA model can more effectively handle background texture occlusion and improve the detection accuracy of small and occluded targets. This approach will provide a more effective means for the management and upkeep of the power system, improve work efficiency, reduce costs, and effectively avoid circuit hazards caused by foreign objects in transmission lines.

The primary contributions of this scholarly work are delineated as follows, taking into account various perspectives:

1. This paper proposes an innovative WSA network that addresses the difficult problems in foreign object detection on power lines and combines the specific advantages of BSAM, improved LSKNet, and bifpn technologies to achieve more efficient transmission line safety detection.
2. The large convolutional structure of LSKNet is introduced, and the improved large convolutional structure of LSKNet exhibits a more extensive input coverage and stronger feature extraction capability that can effectively capture the contextual information and fine-grained features of the target.
3. The BiFPN structure is used to improve the traditional PAN–FPN structure and optimize the weight distribution of the fusion results. By reasonably adjusting the weight distribution of the fusion results, the efficacy and resilience of the network for object identification assignments are additionally enhanced.

## 2 YOLOv8 algorithm

The YOLOv8 detection algorithm is a lightweight anchor-free model that directly predicts the center of extraneous materials within power conduits instead of the offset of the known anchor frame (Talaat and ZainEldin, 2023; Pan et al., 2024). The algorithm can quickly locate the foreign objects in transmission lines to be detected during detection. Anchorless detection reduces the number of box predictions, accelerates non-maximum suppression and replaces C3, the main building block in the network, with C2f, in which all the outputs of the bottlenecks are concatenated. In C3, only the output of the last bottleneck is utilized, and the kernel size of the first convolution is changed from 1 × 1 to 3 × 3, which makes extracting foreign body features of power transmission lines initially more efficient. More information about the characteristics of extraneous materials within power conduits can be obtained.

In the neck part, the features are directly connected without forcing the use of the same channel size, which reduces the number of parameters and the overall size of the tensor so that the volume of the final power line foreign object detection model is also reduced accordingly.

Since YOLOv8 uses a large grid to segment the image, there may be errors in the precise location of foreign objects in power lines, and it may not be suitable for precise positioning application scenarios. Second, YOLOv8 uses a constant scale during training, and the scale of the detection target may change due to factors such as distance and viewing angle. Therefore, in the application scenario, the YOLOv8 network is improved and a multiscale process is used to generate new feature maps to improve the accuracy and reliability of target recognition (Liu et al., 2024; Vahdani and Tian, 2024).

## 3 WSA

To enhance the precision and efficiency of foreign object detection on power transmission lines, this study employs the rapid and accurate YOLOv8 algorithm as a foundation. The YOLOv8 algorithm is renowned for its exceptional speed and detection accuracy; however, it encounters difficulties in identifying targets obscured by background elements. In order to overcome this limitation, a new network structure named Weighted Spatial Attention network (WSA) is proposed based on YOLOv8. The WSA network combines a spatial attention mechanism with a weighted feature pyramid network, creating a complementary effect that significantly improves the detection performance of obscured targets. Additionally, to address the issue of partially occluded targets, this study utilizes data augmentation methods, including random occlusion techniques, to enhance the model's flexibility and robustness in handling partially obscured targets.

**Figure 1:**
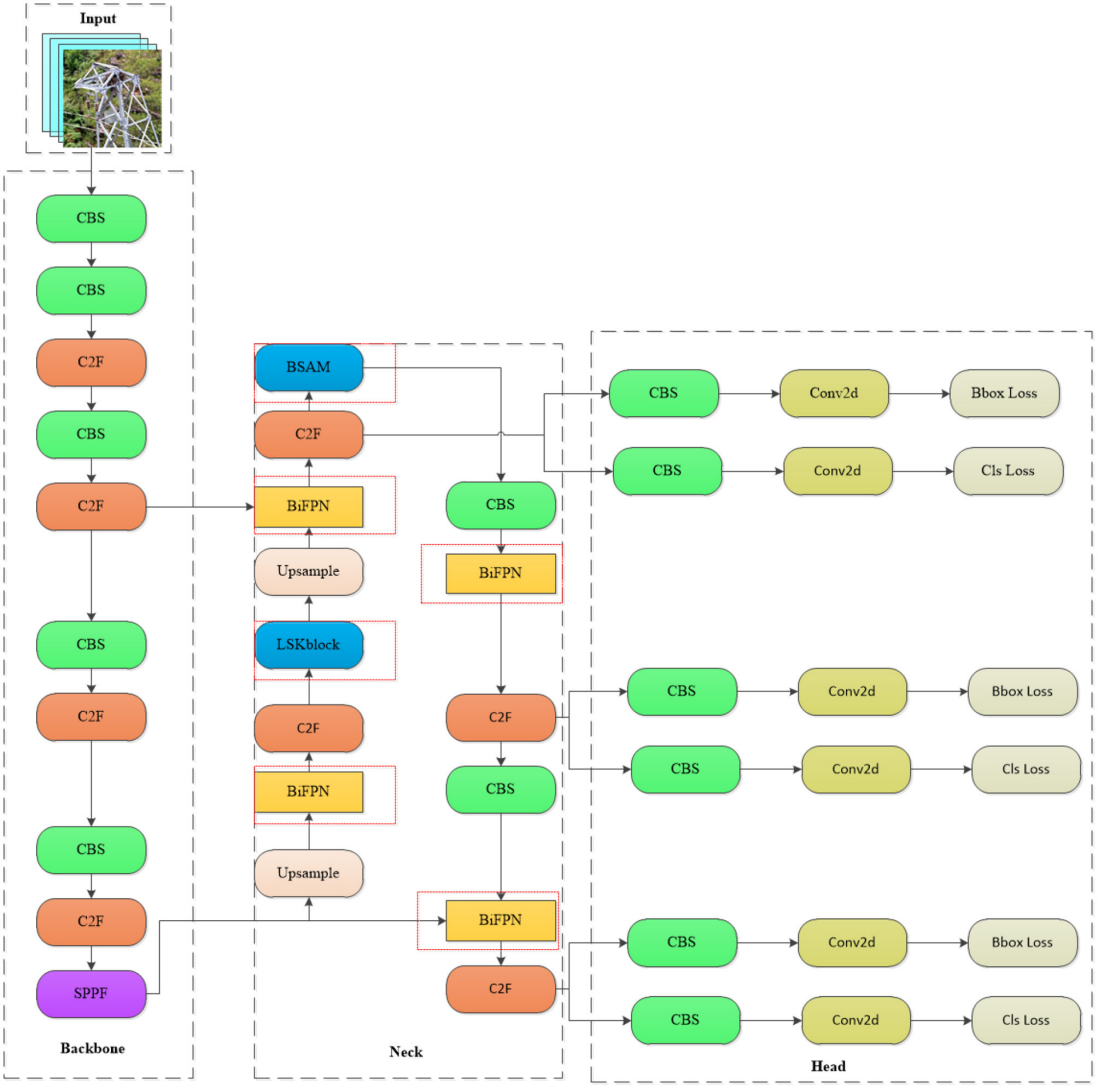
WSA network architecture diagram (in the neck network diagram, the network enclosed by the red dashed lines represents the addition made in this paper, where the LSK block is a modified version of the original module).

By integrating the LSKNet large convolutional framework into the BSAM foundation, the WSA network enhances its capabilities by broadening the receptive field and capturing fine-grained feature details. Furthermore, we have strengthened the traditional PAN-FPN architecture through the implementation of the BiFPN structure and an improved weight allocation method, thereby enhancing the integration of multi-scale features. With the integration of a weighted scheme, the network utilizes information from different feature layers more effectively, enhancing its focusing and discriminative abilities for transmission line detection. The WSA network retains the advantages of existing structures while enhancing the framework's effectiveness and robustness, demonstrating promising applicability and prospects for widespread adoption. The detailed structure of the WSA network architecture is depicted in Figure 1, clearly illustrating how the network achieves efficient identification of foreign objects on power transmission lines through the collaborative work of its various components.

### 3.1 LSKNet

**Figure 2:**
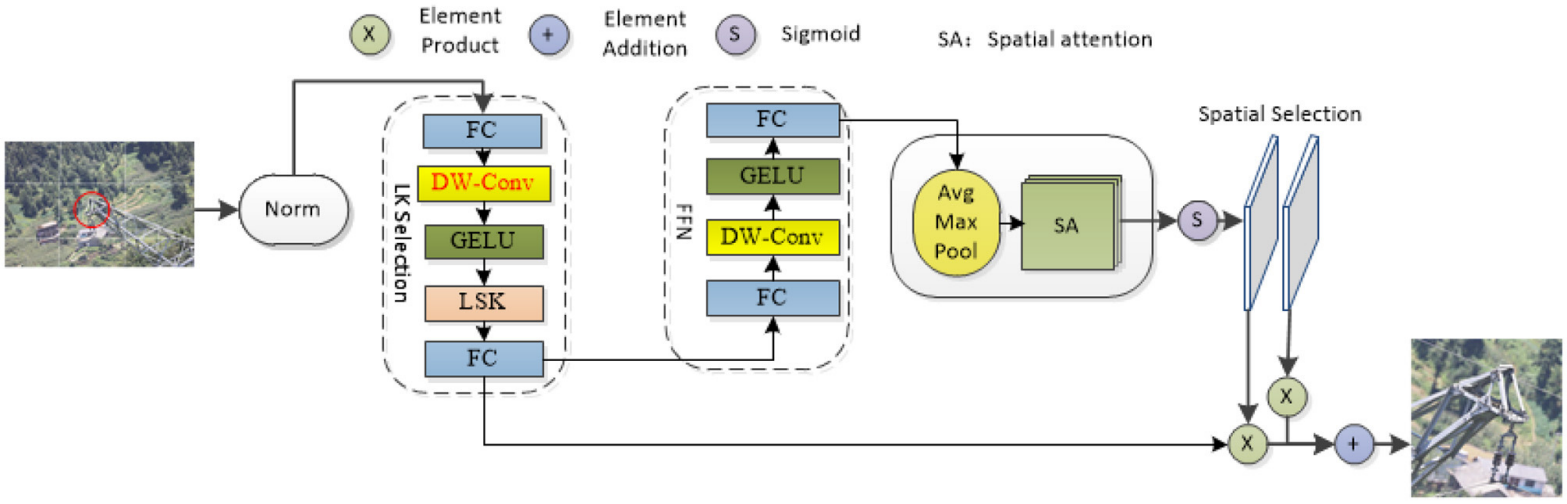
LSK model diagram (in the diagram, the components highlighted in red denote the added depth convolutional blocks, while “Avg Max Pool” refers to the sequential application of average and maximum pooling operations).

LSKNet is a lightweight network proposed by Li that can dynamically adjust the spatial receptive field (Li F. et al., 2023; Li Y. et al., 2023). Most of the images in the transmission line foreign object dataset are obtained by drone aerial photography. The objects in many pictures are small and not easily recognized by the model. The model must rely on their background and surrounding environment to identify these objects. Addressing the issue concerning image detection in intricate surroundings, LSKNet has emerged. Using the rotation-sensitive convolution operation of LSKNet, it can effectively capture the rotation information of extraneous matter on power lines and improve the accuracy of the target detection (Guo et al., 2021; Chen et al., 2023; Hanzl et al., 2023; Ju and Wang, 2023; Kou et al., 2023; Zhang T. et al., 2023). This paper introduces a dynamic receptive field adjustment method based on large kernel selection sub-blocks and adds a deep convolution to the basic LKS election aiming to diminish the parameter count while concurrently enhancing the capacity for characteristic portrayal. In addition, feed-forward network sub-blocks are applied to channel blending and feature expression refinement, additionally, the model's precision in capturing relevant details is significantly improved. By dividing the image into multiple grids and performing feature extraction on each grid and by integrating these features, we empower the system to concentrate its attention more acutely on the area of interest. This approach improves the robustness and generalizability of the model. As shown in Figure 2, red DW-Conv is the added depth convolution.

LSKNet plays a key feature extraction role in foreign object detection on transmission lines. First, LSKNet can dynamically adjust the feeling field of the network, through its core module, the LSK module. The network can adaptively select different sized convolution kernels according to the content of the input image and thus can capture features at different scales. This capability is particularly important for the precise identification of minor objects within satellite imagery, as small targets may require finer features to distinguish. Second, LSKNet uses a spatial selection mechanism to weight the processed feature maps of the convolution kernel at different scales. This mechanism allows the network to spatially incorporate these feature maps, thereby enhancing the contextual understanding of the target surroundings while maintaining computational efficiency. Last, the inclusion of LSKNet was driven by its ability to dynamically adjust the spatial receptive field, which is crucial for identifying small and distant objects common in transmission line imagery. The large convolutional structure of LSKNet provides extensive input coverage and robust feature extraction, enabling the model to effectively capture contextual and fine-grained features of targets.

In practical applications, LSKNet can overcome the effects of light changes, shadows, occlusions, and other factors on the detection results and can accurately identify different types of foreign objects, such as bird nests and kites. This function not only reduces the risk of missed detections and false detections but also improves the reliability of transmission line monitoring.

### 3.2 BSAM attention

**Figure 3:**
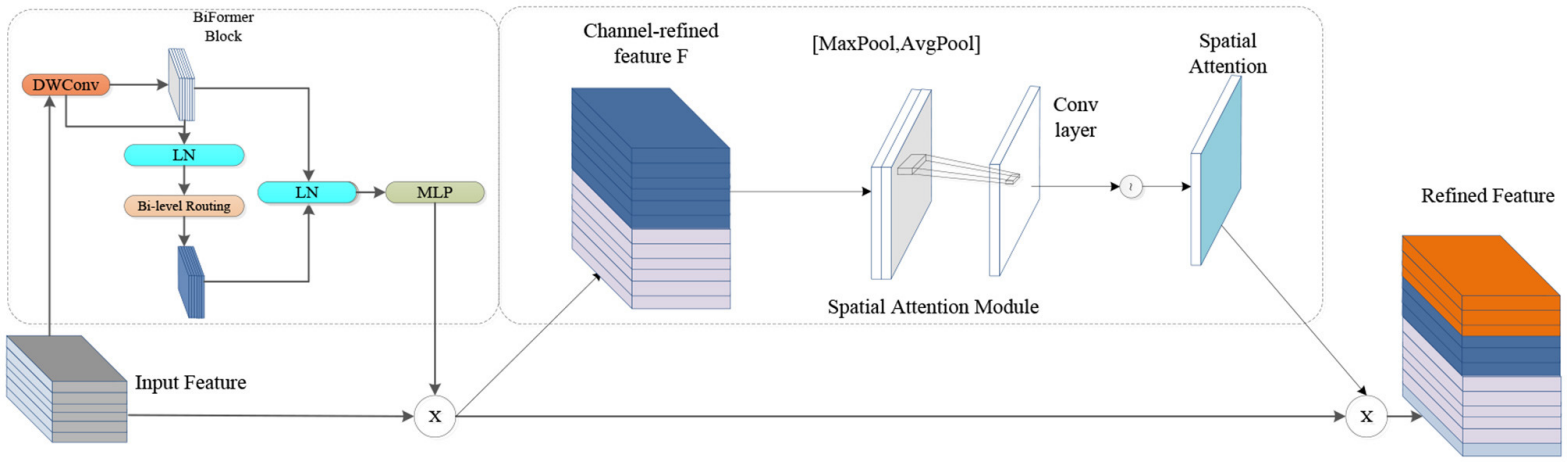
Diagram of the BSAM architecture.

The BSAM is designed to integrate the dual advantages of the CBAM and BiFormer to bolsters the model's capacity to discern targets and pay attention to local details. The CBAM weights the depth and spatial aspects of the feature map through channel and spatial attention mechanisms, respectively, to extract useful feature information. BiFormer uses the internal attention mechanism of the transformer to dynamically learn the long-range dependencies between feature image pixels. By combining these two attention mechanisms, the BSAM can more effectively seize the contextual cues and intricate characteristics of the target (Guo et al., 2023; Zheng et al., 2023; Zhu et al., 2023; Hu et al., 2024a,b). The CBAM consists of two parts: channel attention and spatial attention. The BSAM also inherits this feature. The channel attention mechanism enters the input image feature map into a depth-separable convolution to reduce the amount of calculation and then normalizes it. The layer enters the dual-layer routing attention mechanism, pruning and filtering the feature information, only focusing on the routing area with the most feature information, filtering other areas, and then passing the multilayer perceptron to obtain the deep feature map (Cheng et al., 2023). These feature maps are average pooled and max pooled in units of channels, and then the results are connected, converted to dot products through convolution, and applied to the feature maps according to the spatial attention channel weights. The architecture diagram is shown in Figure 3.

Compared with existing attention mechanisms, the BSAM has more advantages when processing small target images. By integrating a fusion of an introspective focus system with convolution operation, the BSAM can efficiently extract feature information and adapt to different processing methods in different scenarios. The BSAM enhances the model's understanding of the image context by integrating multilevel feature information, which is crucial for the accurate identification and positioning of foreign bodies in complex backgrounds, especially in scenarios such as transmission lines, where foreign bodies may be similar to the background texture or appear at different angles and under different light conditions.

Compared with the addition of CBAM or BiFormer, the number of parameters in the BSAM has not significantly increased, but it can accurately capture the key information in the input data and filter out redundant and noisy features. This ability greatly improves the robustness of the model, allowing it to better adapt to target detection tasks under background texture occlusion and lighting conditions. Similar to the CBAM, the BSAM has the ability to adaptively weight features of different scales, thereby retaining richer semantic information and further improving the generalization ability of the model (Hanning et al., 2023; Li F. et al., 2023; Li K. et al., 2023; Liu L. et al., 2023; Liu X. et al., 2023).

First, the input image features enter a 3 × 3 depth separable convolution, and then the results enter the normalization layer and dual-layer routing attention mechanism module, which divides the feature map *X*∈*R*^*H*×*W*×*C*^ into NxN non-repeating regions. X represents the feature map of the input; H, W and C represent the height, width and number of channels, respectively; each region includes *H*×*W*×*N*^2^ a feature vector, which can be converted to Equation (1); and the query, key, and value tensors Q, K, and V are derived ${\in R}^{N^{2}\times \frac{HW}{N^{2}}\times C}$, which have linear projections such as Equation (2). Only the top-k connections in each region are retained to prune the association graph *I*^*r*^ = *topkIndex* (*A*^*r*^), where *A*^*r*^ is the adjacency matrix of the interregion affinity map, and tensors of keys and values for query tokens in each region are collected. O is the tensor of the output, Attention (⊙) is an attention operation, and LCE (⊙) is a depth convolution parameterization, as shown in Equations (3, 4), where *K*^*g*^, *V*^*g*^ is the tensor of the aggregation key and value.


(1) $$\begin{array}{l} {X^{r}\in R}^{N^{2}\times \frac{HW}{N^{2}}\times C} \end{array}$$

(2) $$\begin{array}{l} Q=X^{r}W^{q},K=X^{r}W^{k},V=X^{r}W^{v\;} \end{array}$$

(3) $$\begin{array}{l} {K^{g}=gather\left(K,I^{r}\right),\;V}^{g}=gather\left(V,I^{r}\right) \end{array}$$

(4) $$\begin{array}{l} O=Attention\left({Q,K}^{g},V^{g}\right)+LCE\left(V\right) \end{array}$$

Second, the module dedicated to spatial focus is activated, following which average and maximum pooling steps are applied, the feature maps generated by them are spliced, and the convolution operation is applied to the spliced feature maps to generate the final feature map. The whole process in Equation (5) is expressed as follows: *f*^7 × 7^ represents a 7 × 7 convolution operation, *MaxPool, AvgPool* represents the maximum pooling with the mean pooling, and *M*_*s*_ represents the spatial attention module.


(5) $$M_{s}\left(O\right)=\sigma (f^{7\times 7}([AvgPool\left(F\right);MaxPool(F)])$$

The BSAM attention mechanism shows significant advantages and effects for identifying unauthorized items within the framework of power conduits. This combined attention mechanism fully utilizes the characteristics of the channel attention and spatial attention of the CBAM and weighted double-layer path attention mechanism of BiFormer. This approach elevates the model's skill in handling capture image features and focus attention.

First, by focusing on key areas in the image through ensemble channel attention and spatial attention mechanisms, important spatial locations in the image can be identified, and more attention forces can be assigned to these areas to highlight the target object in a complex background. This approach improves the detection accuracy of the model under background texture occlusion and reduces false detections and missed detections. Second, the weighted dual-layer path attention mechanism of BiFormer allows the model to focus on different parts of the image at different levels and integrate multilevel feature information. This mechanism enhances the model's understanding of the contextual information and spatial layout of the image, more accurately identifying and locating foreign objects. By integrating channel attention and spatial attention with a weighted dual-layer path attention mechanism, this mechanism can more comprehensively focus on all aspects of the image, including channel dimensions, spatial dimensions, and different levels of feature information.

### 3.3 BiFPN

Another innovative point of this article is to introduce a weighted feature pyramid structure called BiFPN to solve the problem of low detection accuracy caused by light intensity. The BiFPN structure is able to grasp the significance of various input characteristics, the process involves iteratively conducting a hierarchical integration of coarse-to-fine and fine-to-coarse multi-tier feature merging, thereby seamlessly blending features across disparate scales (Liu L. et al., 2023). In traditional feature pyramid networks, feature fusion is usually accomplished by simple summation or averaging operations, which may lead to the loss of information. BiFPN controls the fusion of features at different levels through learnable weights, thus achieving lossless fusion of features while retaining more useful information, enhancing the model's perception capabilities and accuracy.

**Figure 4:**
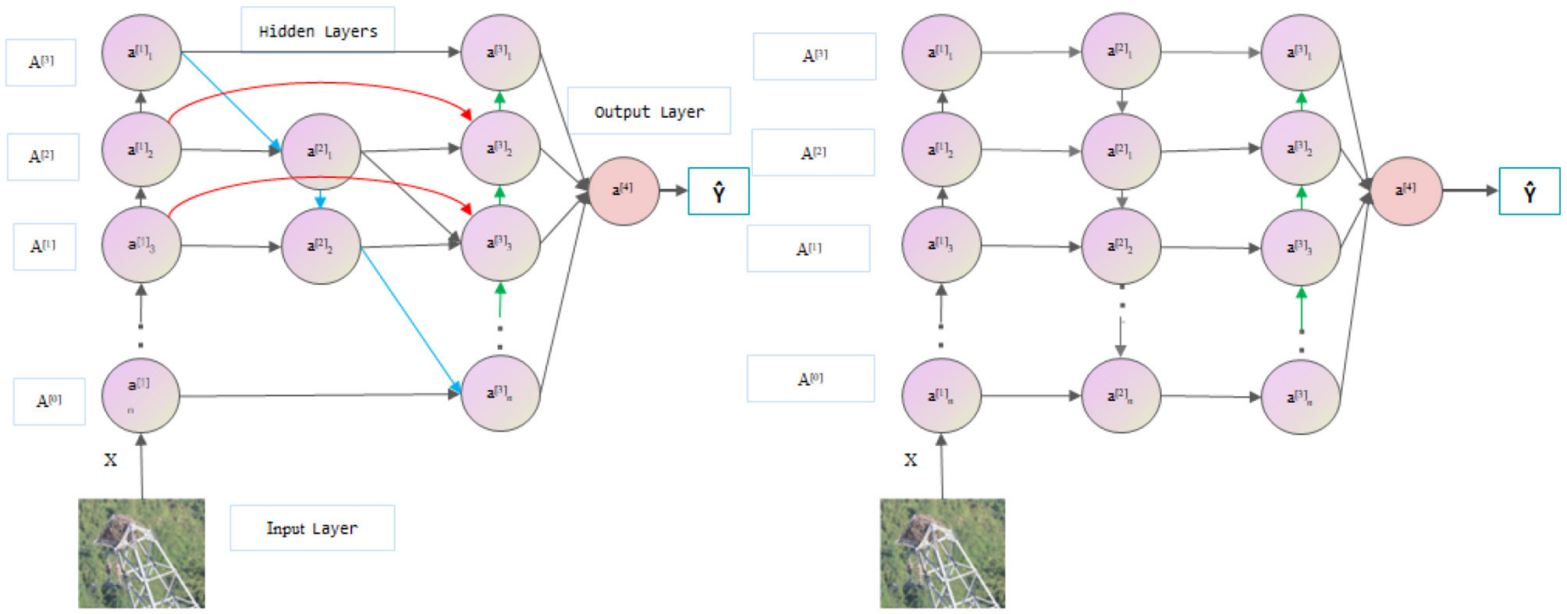
Comparison of BiFPN **(left)** with the PANet **(right)** network (the green arrows represent upsampling, the blue arrows represent downsampling, and the red arrows represent skip connections).

In the YOLOv8 model, the PANet pyramid structure is used to enhance the receptive field of the network. Compared with the original FPN pyramid, PANet has more bottom-up path aggregation but only one input, and there is no feature fusion. Therefore, in the BiFPN module, we remove PANet but still retain its output and realize the transfer of information by connecting the previous node to the next node. In addition, BiFPN also adds skip connections based on PANet to fuse the previous features. As shown in Figure 4, each circular block is image feature information extracted by convolution. Multiple BiFPN modules are applied to the entire YOLOv8 structure to fully integrate features and better utilize different levels of semantic information (Wang et al., 2019; Chen et al., 2021; Islam et al., 2023; Wu et al., 2023).

When performing feature fusion, different feature inputs have different resolutions and different output contributions to the feature network, so the network needs to learn the weights. However, general weighted fusion is not restricted, and *w*_*i*_ makes the training process difficult to stabilize. It is not easy to converge, so BiFPN uses a fast normalized feature fusion operation, as shown in Equation (6):


(6) $$\begin{array}{l} O=\Sigma_{i}\frac{w_{i}}{\varepsilon +\Sigma_{j}^{w_{j}}}I_{i} \end{array}$$

First, the activation function *w*_*i*_≥0 ReLU is used to ensure that it is not <0. Second, a fixed value for ε, generally 0.0001, is used to ensure stable training. This approach avoids the Softmax operation and is faster than using Softmax in training. There is a significant improvement, and the final feature fusion network will also be input from bottom to top, thus constructing a weighted, two-way feature pyramid network.

Within the YOLOv8 framework, the integration of BiFPN enhances the capability of inter-scale feature interaction, which is particularly beneficial for bolstering detection capabilities in complex environments, such as power transmission lines. By establishing lateral connections that allow for direct fusion of features across various layers, BiFPN ensures a rich and detailed representation capturing both macro and micro aspects of the scene. This is crucial for the identification of foreign objects that may appear in various sizes and shapes against diverse backgrounds.

Furthermore, BiFPN's architecture is designed to iteratively refine these feature representations, enabling the model to progressively improve its understanding of the spatial hierarchy within the imagery. This refinement process is essential for the accurate localization and classification of foreign objects, especially when they are obscured or camouflaged by the natural setting of the power lines. Essentially, BiFPN contributes to the YOLOv8 model in two significant ways: it enriches the feature hierarchy through its bidirectional fusion process and enhances the model's ability to discern and locate foreign objects with high fidelity. This dual enhancement translates to a marked improvement in the detection accuracy of foreign objects on power transmission lines, thereby strengthening the model's applicability in real-world scenarios.

## 4 Research methodology and experimental evaluation

### 4.1 Dataset construction

To address the scarcity of large-scale public datasets in the field of foreign object detection on power transmission lines, this study employed a dataset synthesized from aerial imagery captured by inspection drones and images collected from the internet, enhanced through various data augmentation techniques. Initially, web crawling technology was utilized to search for and download images related to foreign objects on power transmission lines from search engines such as Baidu and Google. Subsequently, a portion of the images was obtained through drone photography. However, due to environmental constraints, the number of images captured in this manner was limited.

To overcome this limitation and to create a more complex and diverse dataset, several data augmentation strategies were implemented. Standard enhancement methods, such as image flipping and rotation, were applied. In addition, a random occlusion technique was employed, where parts of the images were masked with black to simulate complex environmental backgrounds. Building on this, image synthesis techniques were used to place foreign objects onto power lines as a special form of dataset expansion.

The resulting dataset, comprising 2,300 images, is named the PL dataset in this paper. It mainly includes four distinct categories: bird nests, kites, balloons, and debris attached to transmission lines. Labeling was facilitated by the use of the third-party library Labeling and semi-automated annotation from Make Sense, which aids in the accurate localization and identification of foreign objects in the images.

Furthermore, the dataset was meticulously divided into training, validation, and testing sets in a ratio of 8:1:1, respectively. The training set, consisting of 1,840 images, provides a substantial amount of data for model learning. The validation and testing sets, each containing 230 images, are used to assess model performance and generalization capabilities. This partitioning strategy ensures a robust evaluation of the model's effectiveness in detecting foreign objects on power transmission lines.

### 4.2 Training configuration

The corresponding parameters of the model are shown in Table 1. The test hardware platform environment is Python3.8 and CUDA11.3, the GPU graphics card is an NVIDIA RTX3090, and the memory is 43 GB. The label smoothing value is set to 0.005. The learning rate was obtained using the cosine annealing algorithm. The maximum learning rate is set to 0.01, and the minimum learning rate is set to 0. The model gradually reduces the learning rate in the form of a cosine function according to the number of iterations during the training process. The training process is divided into multiple cycles. In each cycle, the learning rate starts from the initial value. As the number of iterations increases, it gradually decreases to a smaller value according to the curve of the cosine function and then starts again in the next cycle. This adjustment method not only ensures that the model can quickly converge in the early stages of training but also maintains a small learning rate in the later stages of training to prevent the model from oscillating near the optimal solution. The cosine annealing formula is shown in Equation (7), where lr represents the current learning rate, *lr*_−_max represents the maximum learning rate, *e*_−_*total* represents the total number of training rounds, and *e*_*now* represents the current training round.


(7) $$\begin{array}{l} lr=\frac{1}{2}lr_{-}max\times \left(1+cos\left(\frac{e\text{\_}now\times \pi }{e_{-}total}\right)\right) \end{array}$$

**Table 1:** Model parameter setting.

**Parameter name**	**Parameter value**
Imgsz	640^*^640
Epoch	300
Batch size	32
Lr_max	0.01
Optimizer	Adam

### 4.3 Analysis of model performance indicators

This paper uses the average precision mean, precision rate, recall rate, FPS and parameter quantity indicators to test the model performance (Du et al., 2023; Li F. et al., 2023; Vidit et al., 2023; Wang et al., 2023a,b; Lin et al., 2024). The mAP is the average accuracy of each category of detection results. AP refers to the area of the curve surrounded by the horizontal axis and vertical axis of the precision rate and recall rate, respectively. The calculation formulas for precision and recall are shown in Equations (8, 9), respectively.


(8) $$\begin{array}{l} Recall=\frac{TP}{TP+FN} \end{array}$$

(9) $$\begin{array}{l} Precision=\frac{TP}{TP+FP} \end{array}$$

In the formula, TP is the number of correctly identified targets. Generally, when the IoU threshold is ≥0.5, it is considered to be a correctly identified target. FP is the number of incorrectly identified targets; FN is the number of missed targets; Precision is the number of targets in the model, the proportion of correct targets among the detected targets; and Recall is the proportion of targets correctly identified by the model among the total number of all real targets (Sun et al., 2024). The number of detected categories in this article is 4; the mAP is shown in Equation (10).


(10) $$\begin{array}{l} mAP=\frac{1}{4}\overset{4}{\underset{i=0}{\sum }}AP_{i} \end{array}$$

### 4.4 Comparison experiment

This paper introduces the incorporation of depthwise convolution into the existing LSKNet network, with the improved results presented in Table 2. For ease of reference, Table 2 only displays the comparison with LSKNet-D, which is still referred to as the LSKNet network.

**Table 2:** Large kernel selection subblocks with added deep convolution contrast.

**Model**	**P/%**	**R/%**	**mAP/%**	**Parameters**	**FPS (f/s)**
LSKNet	87.3	90.7	93.4	3349118	212.65
AFPN	89.5	83.8	89.9	2328193	262.54
BasicRFB	91.1	81.0	91.2	3446020	234.21
Botnet	92.7	85.9	92.0	5695276	212.15
C2f_repghost	87.1	83.4	90.2	4240460	204.47
C2f_scconv	86.8	85.4	90.8	2617900	233.45
**LSKNet-D**	**86.6**	**91.2**	**92.6**	**3307469**	**234.29**

The bolded values represent the experimental results of the research network presented in this article.

The data in Table 2 indicate that the Botnet model exhibits the highest precision, reaching 92.7%, but it also has the largest number of parameters, suggesting a more complex model. On the other hand, the AFPN model performs poorly in recall, with only 83.8%, yet it has the highest frames per second (FPS), reaching 262.54 FPS, indicating an advantage in speed. The C2f_repghost model has a slightly lower precision but the second-largest number of parameters, whereas the LSKNet-D model achieves the highest mean Average Precision (mAP) among all models, with 92.6%, while maintaining a relatively high FPS. The addition of depthwise convolutional layers resulted in a 0.8% decrease in mAP for the LSKNet model, which may indicate that the inclusion of depthwise convolutional layers could make it more challenging for the model to fully learn effective feature representations during training. However, with a 1.2% reduction in the number of parameters, the increase in FPS by 10.1% suggests that LSKNet can achieve rapid object detection at a lower computational cost, suitable for devices with limited resources. For instance, operating on drones significantly expands the application scope of LSKNet in the field of foreign object detection on power transmission lines.

**Figure 5:**
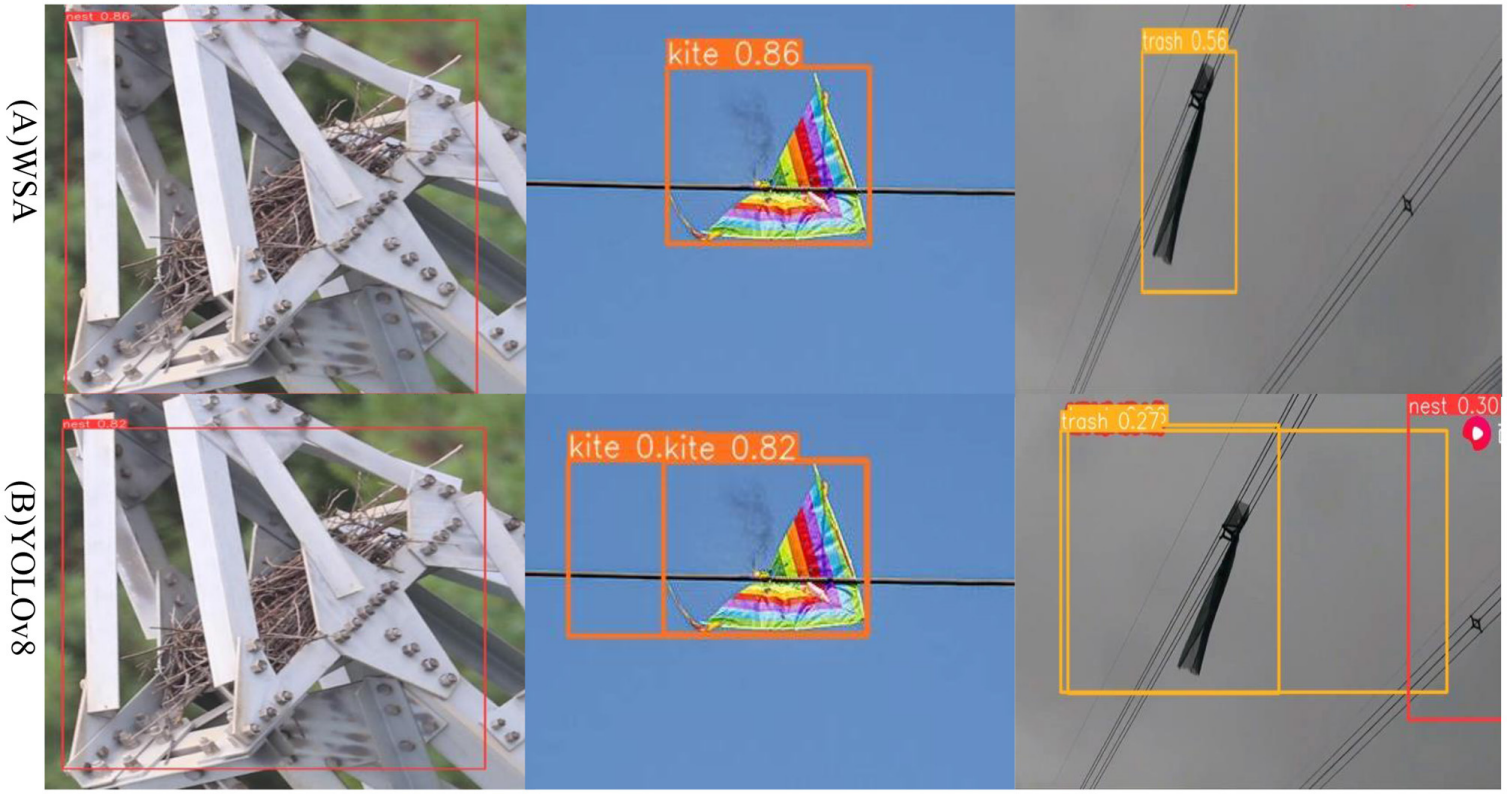
WSA and YOLOv8 algorithms for the identification results, **(A)** represents the detection effect of the WSA algorithm, **(B)** represents the detection effect of the YOLOv8.

Figure 5 shows the detection results for foreign objects such as bird nests and kites parked or attached to transmission lines. In Figure 5B, YOLOv8 recognized the spacer as a bird's nest, and the garbage recognition accuracy was only 27%. However, in Figure 5A, the WSA achieved a garbage recognition accuracy of 56%, and there were no false detections. Compared with YOLOv8, the WSA model has improved image detection accuracy and can reduce the occurrence of false detections. Thus, the WSA has higher reliability and accuracy in practical applications, which is beneficial for resource-constrained environments. Deployment and application in an environment can better meet the demand for a balance between the target detection effect and computing resource consumption.

However, the WSA model may also misdetect foreign objects in transmission lines during detection. First, the WSA model may not have sufficient recognition capabilities for certain types of targets, which may be attributable to the number of targets of that type in the training data caused by a low or uneven distribution. In addition, the target detection algorithm may have certain limitations on changes in the scale, posture, shape, etc., of the target, which may also cause some targets to fail to be accurately detected. Second, the WSA model may not perform well in difficult situations such as background texture occlusion, low light conditions, or occlusion. Especially when there are many distractors or when the target is highly similar to the background, the algorithm may not be able to accurately locate and identify targets. If the target is partially or completely occluded, the algorithm may not be able to obtain enough information for accurate detection. Moreover, the parameter settings and model selection in the algorithm may also affect the results of target detection. If some parameter settings are unreasonable or the model selection is not suitable for the current application scenario, target detection may fail or be missed. Therefore, when using the WSA model for target detection, it is necessary to carefully adjust the parameters and select a suitable model to improve the performance and robustness of the algorithm.

**Figure 6:**
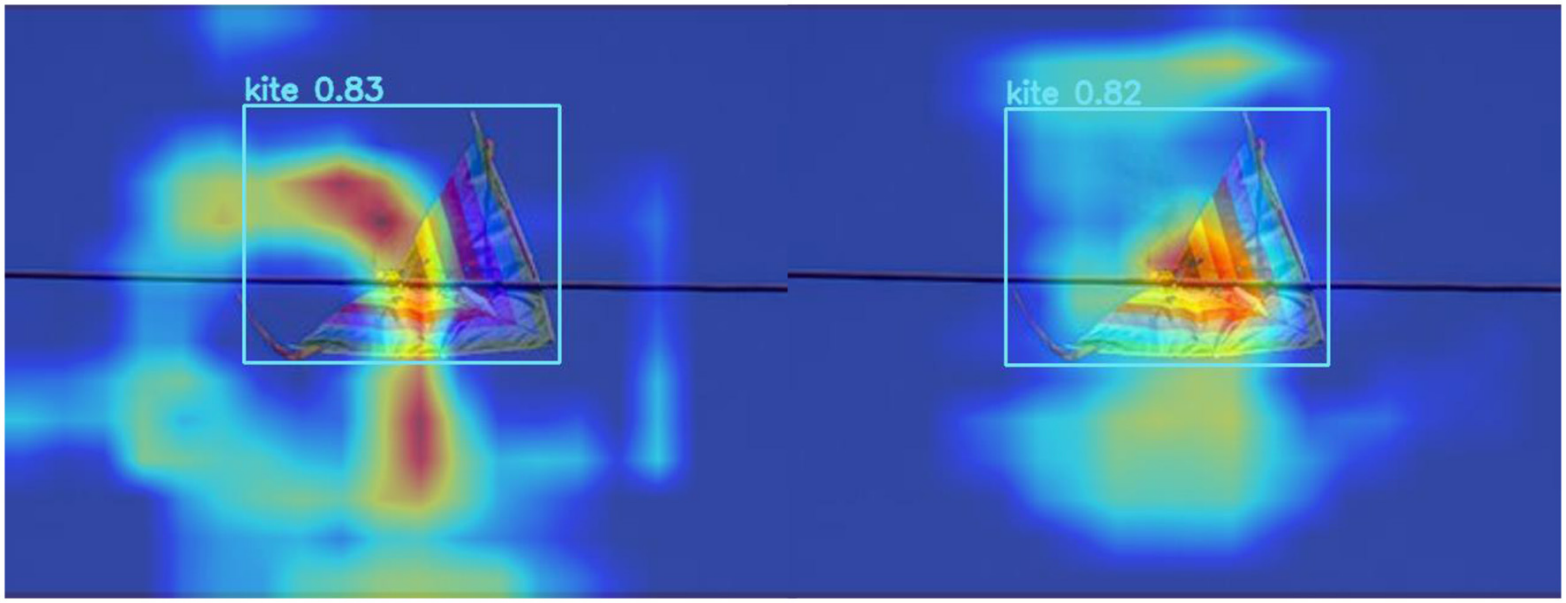
Comparison of thermal maps in layer 12 of the network (on the left side are the outcomes of the WSA model, while the results of the YOLOv8 are presented on the right side).

**Figure 7:**
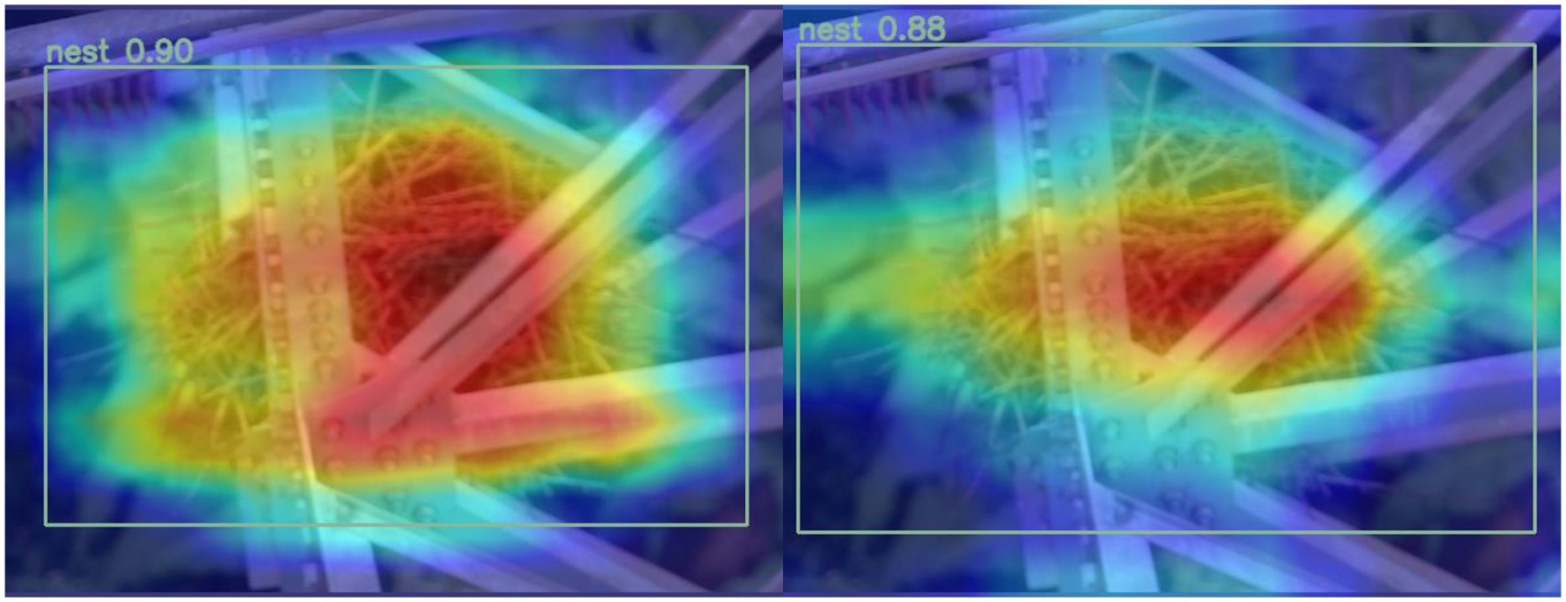
Comparison of thermal maps in layer 14 of the network (on the left side are the outcomes of the WSA model, while the results of the YOLOv8 are presented on the right side).

Figures 6, 7 represent the thermal maps of the improved network (left) and original network (right), respectively. A heatmap is a tool for visualizing the position and confidence of a target object. By observing the thermal maps in Figures 6, 7, we can intuitively observe the difference between the improved algorithm and the original algorithm in the target detection of layers 12 and 14 of the network. The heatmap shows that the improved network has a slightly greater effect than the original network in the same layer and more accurately identifies the target position.

The heatmap can only reflect the detection effect of the algorithm in a certain layer but cannot fully reflect the performance of the entire algorithm. Therefore, this article presents multilayer comparisons and two-layer comparison heatmaps, but more experiments and evaluations are still needed to verify the superiority and feasibility of the improved algorithm. Such multilayer comparison can more comprehensively demonstrate the performance of the improved algorithm and provide a more sufficient demonstration and experimental basis for this article (Li F. et al., 2023; Li G. et al., 2023; Lue et al., 2023; Zhang T. et al., 2023; Zhang X. P. et al., 2023).

The comparative experiments detailed in this article, as depicted in Table 3, serve to underscore the performance metrics of our proposed model alongside established benchmarks in the field of object detection. Acknowledging the stochastic nature inherent to deep learning experiments, we have taken a meticulous approach to ensure the veracity and reliability of our results. To mitigate the effects of variability, we conducted a series of three independent experiments on the YOLOv8 model and other contemporary models, calculating the average mAP @0.5 to provide a consistent and dependable measure of performance.

**Table 3:** Model contrast experiment.

**Model**	**P/%**	**R/%**	**mAP/%**	**Parameters**	**FPS (f/s)**
YOLOv5-n	91.2	84.4	92.2	2806221	254.42
YOLOv5-s	87.4	87.9	92.7	9123740	243.51
YOLOv5-m	91.2	85.2	92.4	25067452	221.54
YOLOv7-n	85.7	86.2	90.9	3152426	249.00
YOLOv6-n	90.4	88.9	91.3	4500080	253.41
YOLOv6-s	87.9	85.4	93.7	16306620	246.15
YOLOv6-m	89.5	86.5	93.1	51998380	254.29
SSD	86.4	89.6	89.5	10014672	201.96
YOLOv9-C	89.8	90.7	89.6	60804152	266.20
Gold-YOLO	91.5	90.7	93.6	6015903	262.54
YOLOX	85.2	93.2	91.1	2617900	249.36
YOLOv8 (Sophia)	87.1	84.7	91.2	3001853	212.86
YOLOv8n (Adam)	92.3	86.4	94.3	3011628	255.75
YOLOv8s (Adam)	91.2	95.6	95.6	11137148	276.43
YOLOv8m (Adam)	89.9	94.2	94.4	25858636	271.22
**WSA**	**97.8**	**92.5**	**97.6**	**3249024**	**269.41**

The bolded values represent the experimental results of the research network presented in this article.

In our quest to optimize the training process, we incorporated the Sophia optimizer (Liu H. et al., 2023), a novel development from Stanford University, into our experimental framework. The Sophia optimizer demonstrated a compelling advantage in terms of training speed, outpacing the Adam optimizer by nearly 1.3 times. This acceleration is attributed to Sophia's refined adaptability to the non-uniformity of different parameter sizes, navigating the curvature of the loss landscape more effectively and converging in fewer iterations.

However, this expedited convergence came with a noted trade-off in accuracy, with a discernible decrease of ~3% points in mAP. This reduction suggests that while Sophia is adept at rapid convergence, it may not consistently reach the global optimum, potentially overlooking solutions that could offer higher accuracy.

In light of these insights, we have retained the Adam optimizer as our baseline model. Adam's balanced approach, offering a commendable blend of speed and accuracy, ensures that our models achieve convergence to solutions that are not only computationally efficient but also align with the high standards of accuracy required for practical applications.

The inclusion of the WSA model in our comparative analysis reveals its exceptional performance, with the highest precision, recall, and mAP among all evaluated models. This outcome highlights the efficacy of the WSA model in achieving a remarkable balance between speed and accuracy, evidenced by its high FPS and reduced parameter count. The WSA model's enhanced performance is particularly noteworthy for its applicability in scenarios with limited computational resources, such as real-time object detection on drones patrolling power transmission lines.

### 4.5 Ablation experiment

Table 4 presents a detailed account of the ablation study results for the key components of our model, which are crucial for assessing the contribution of each module to the overall performance. Conducted with the YOLOv8 network as the baseline model, the ablation study involved enabling or disabling the LSKNet, BSAM, and BiFPN modules individually, providing an in-depth understanding of the role and impact of each module.

**Table 4:** Model ablation experiment.

**Cos_lr**	**LSKNet**	**BSAM**	**BiFPN**	**P/%**	**R/%**	**mAP/%**	**Parameters**	**FPS(f/s)**
**√**	×	×	×	92.3	86.4	94.3	3011628	255.75
**√**	√	×	×	86.6	91.2	92.6	3307469	234.29
**√**	×	√	×	93.7	92.8	95.5	2681304	298.21
**√**	×	×	√	90.7	89.6	94.8	3012524	279.89
**√**	√	√	×	93.6	91.5	94.9	3289752	266.32
**√**	√	×	√	97.5	93.7	97.4	2930462	259.97
**√**	×	√	√	93.1	96.7	97.4	2680408	274.36
×	×	×	×	92.6	88.3	93.6	3011628	289.50
×	√	×	×	84.9	89.5	90.5	3307469	256.19
×	×	√	×	91.8	93.4	96.1	2681304	287.34
×	×	×	√	90.2	90.3	94.9	3012524	238.37
**√**	**√**	**√**	**√**	**97.8**	**92.5**	**97.6**	**3249024**	**269.41**

The bolded values represent the experimental results of the research network presented in this article.

From the table, it is evident that when all modules are inactive (LSKNet, BSAM, and BiFPN are all marked with “ × ”), the model's precision (P/%), recall (R/%), and mean Average Precision (mAP/%) are 92.3%, 86.4%, and 94.3%, respectively, establishing a baseline for model performance. When the LSKNet module is introduced alone, precision drops to 86.6%, suggesting that LSKNet may be incompatible with certain characteristics of YOLOv8 when used in isolation or may have limitations on specific tasks. CosineAnnealingLR (Cos_lr) emonstrates a positive impact on enhancing the precision and recall rates across these models. By optimizing the learning rate schedule, it aids models in converging more rapidly, thereby improving overall model performance.

However, when the BSAM and BiFPN modules are applied individually (third and fifth rows, respectively), an improvement in accuracy is observed, indicating that these modules can independently enhance the model's feature extraction and multi-scale+‘ feature integration capabilities. Notably, the BSAM module, when used alone, significantly increases recall to 92.8% and raises mAP to 95.5%.

Further, when these modules are combined, a significant enhancement in performance is noted. For instance, when LSKNet and BiFPN are used without BSAM (fourth row), mAP reaches 94.8%. When LSKNet is combined with BSAM (second and sixth rows), a balanced improvement in precision and recall is observed, with mAP reaching 94.9%.

Most strikingly, when all three modules are fully integrated (last row), the model achieves an outstanding mAP of 97.6%, a 3.3 percentage point improvement over the baseline model. This result not only demonstrates the complementarity of the modules but also the effectiveness of the integration strategy.

Although there is a 7.8% increase in the number of parameters, this increase is justified considering the significant performance improvement. Particularly in the task of foreign object detection on power transmission lines, the model has shown strong detection capabilities, achieving accurate and stable detection results even under background texture occlusion and varying lighting conditions.

### 4.6 Result analysis

**Figure 8:**
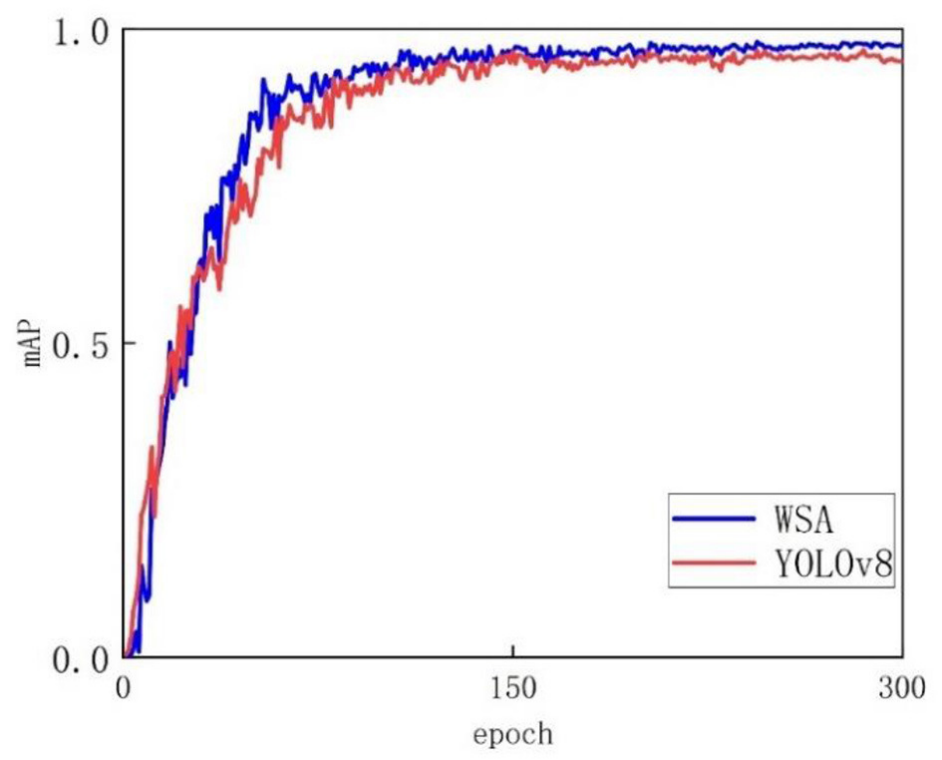
Accuracy change curve of the validation set.

Figure 8 shows the experimental results, where the horizontal axis represents the number of training rounds and the vertical axis represents the accuracy of the mAP50. Figure 8 clearly shows that during the training process, as the number of iterations gradually increases, the performance of the WSA model gradually improves and eventually reaches a stable state. In terms of accuracy, the WSA model shows significant advantages, and its performance is significantly better than that of the YOLOv8 model. Whether using a short training step size or a long training step size, the WSA model is able to maintain a relatively high accuracy level, showing its strong stability and robustness. In contrast, the accuracy of the YOLOv8 model appears to be more variable, and its overall accuracy is lower than that of the WSA model. This advantage mainly stems from the unique structural design of the WSA model. Through a carefully designed network structure and algorithm optimization, the WSA model can more effectively extract key features and make more accurate predictions when processing complex data.

**Figure 9:**
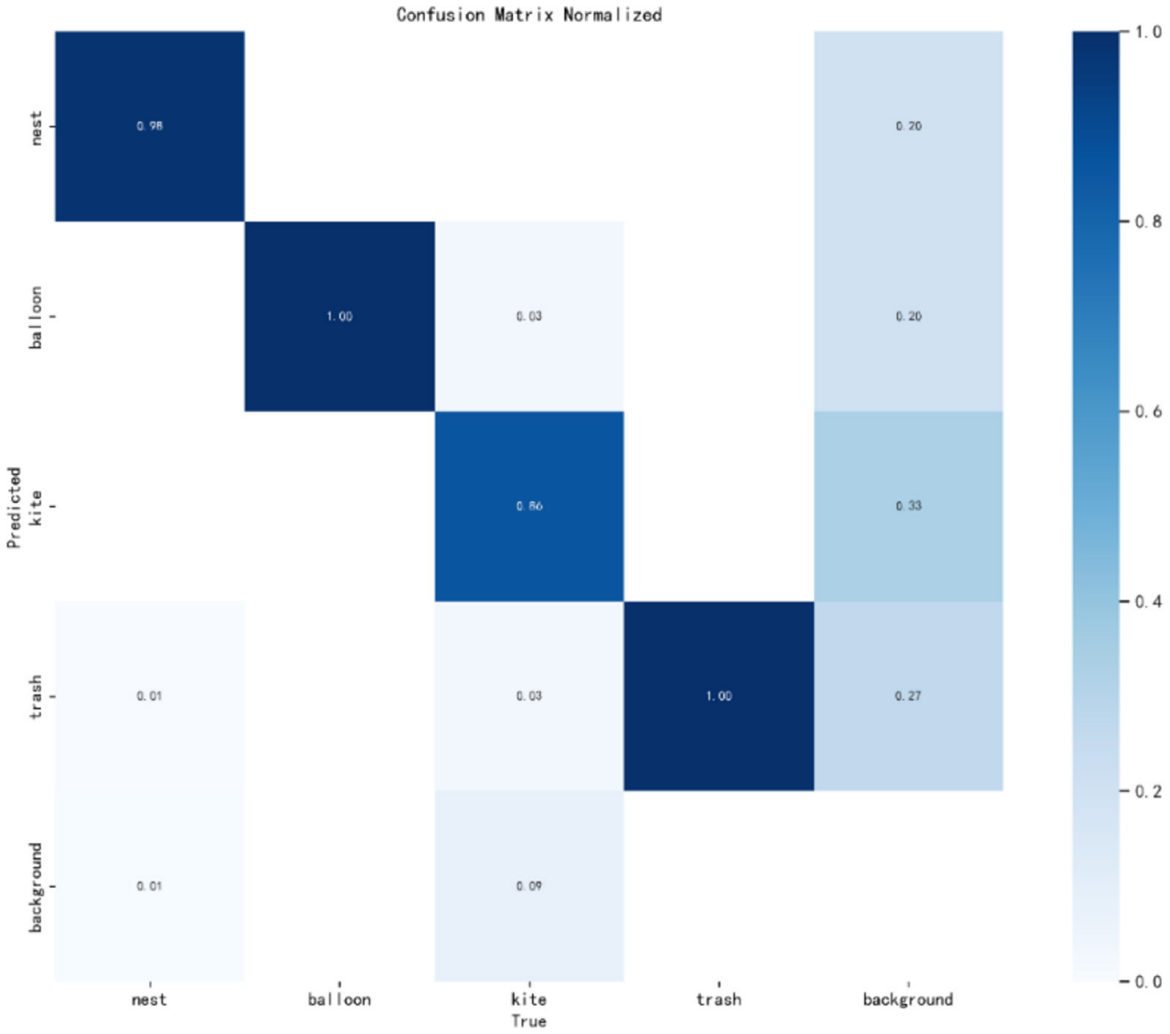
Normalized WSA confusion matrix.

Figure 9 presents the normalized confusion matrix from our test on the PL dataset, offering insights into the model's performance across different categories. The matrix is structured such that rows indicate the predicted categories, while columns represent the actual categories. Each cell's value within the matrix corresponds to the proportion of predictions for that category. Diagonal values signify correct predictions, whereas off-diagonal values denote incorrect ones. Despite an overall accuracy of 97.6%, we identified a specific shortfall in the detection of kites, with an accuracy of only 86% and a notably high false negative rate.

Upon meticulous review, the primary cause of this issue was attributed to the dataset's sample distribution. The kite category in the PL dataset is significantly underrepresented, hindering the model's ability to learn distinguishing features during training. This underrepresentation leads to a model that is less sensitive to kite characteristics, resulting in the observed misdetections.

**Figure 10:**
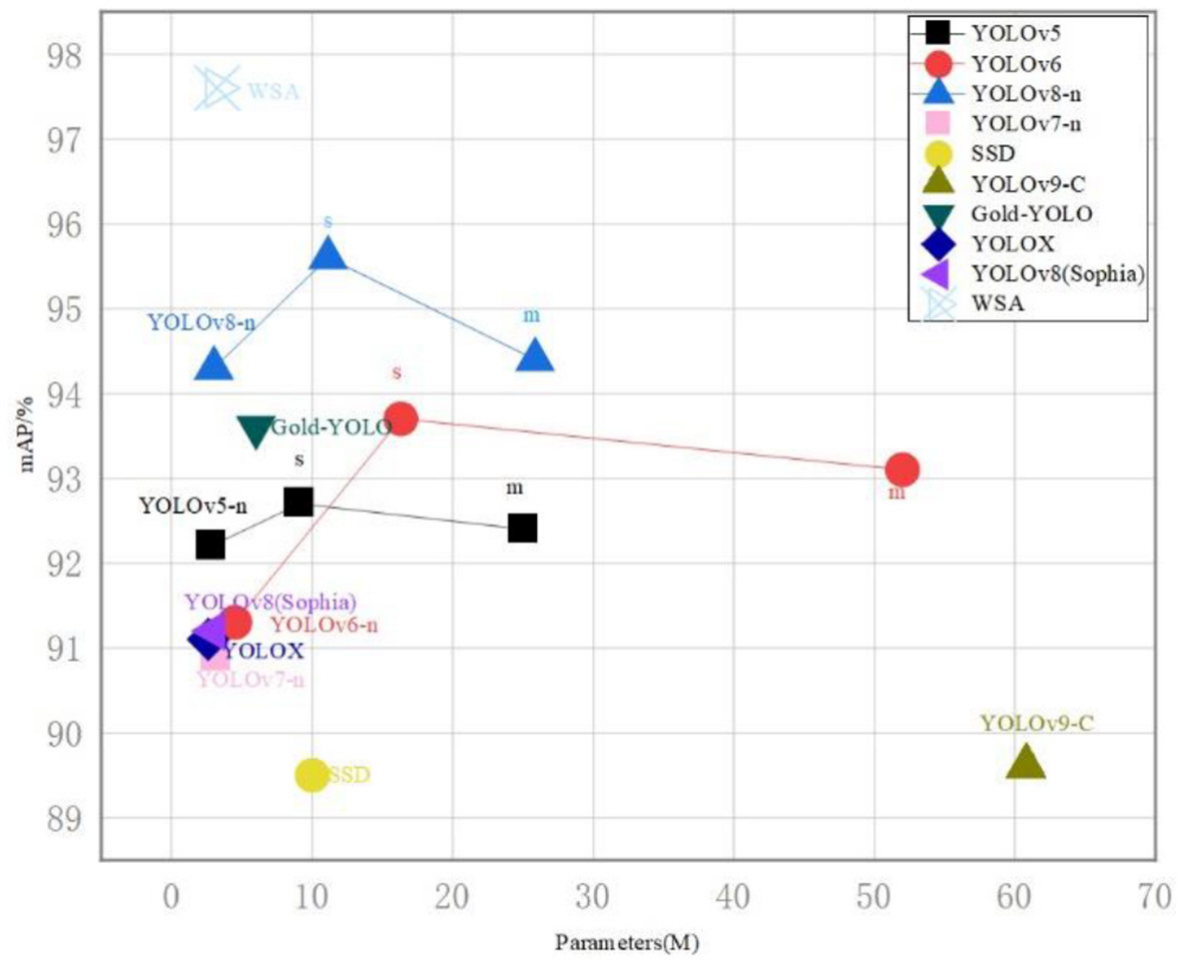
On mobile platforms, the dimensional precision trajectory of streamlined models, employed for comparative analysis with WSA and a spectrum of cutting-edge object detection algorithms.

To address this, we plan to incorporate cost-sensitive learning techniques, which will prioritize underrepresented categories by adjusting the model's focus through weighted sampling. This approach will encourage the model to pay more attention to less frequent but critical classes, such as kites, during the learning process. Additionally, we will explore ensemble learning methods that aggregate the predictions from multiple models. This strategy can improve the final recognition accuracy by leveraging the strengths of various models to compensate for individual weaknesses. See also Figure 10, we provide a comparative analysis of parameter count and dataset accuracy between the WSA model and other state-of-the-art networks. It is evident that the WSA model maintains a comparable number of parameters while achieving a significant boost in accuracy. This observation underscores the efficiency and effectiveness of our model. The WSA model's performance enhancement without a substantial increase in parameters suggests an optimized balance between complexity and accuracy, which is particularly advantageous for deployment in resource-constrained environments.

## 5 Conclusions

This study presents an innovative WSA network model, specifically designed to address the challenge of detecting foreign objects on transmission lines within complex environmental backgrounds. The WSA model integrates a novel weighting mechanism that enables multilevel fusion of feature information and acquisition of global context information, thereby enhancing the detection accuracy in scenarios affected by background texture occlusion and varying light conditions.

Our constructed image dataset, comprising common foreign objects found on transmission lines, served as a robust foundation for model training and validation. The empirical results demonstrate a significant enhancement in the recall rate by 6.1% compared to the existing YOLOv8 algorithm, underscoring the WSA network's superior performance. Moreover, the network's strong hardware adaptability and compatibility suggest its potential for deployment across diverse target detection tasks and datasets.

The ablation study conducted further substantiates the contribution of the three core modules in bolstering the algorithm's performance. However, it is essential to recognize that while the improved algorithm has achieved a 3% increase in accuracy for detecting foreign objects in complex environments, there are areas that require further refinement.

Firstly, the improved model's accuracy, though enhanced, may still be considered marginally insufficient for applications demanding extremely high precision. This limitation could impact the model's applicability in high-stakes scenarios where minute errors can lead to significant consequences.

Secondly, the expansion of the network structure, while beneficial for performance, may introduce a performance bottleneck when deployed on hardware with limited resources. This trade-off between accuracy and computational efficiency is a critical consideration for real-world deployment, particularly in resource-constrained environments.

Lastly, the algorithm's generalizability across different types of foreign body targets may be limited. The WSA network model has demonstrated significant advancements in the detection of foreign objects on transmission lines, offering improved accuracy and efficiency. However, the practical application of this model in real-world scenarios remains to be thoroughly validated. Future research should prioritize the testing and adaptation of the model in actual operational environments, accounting for variables such as diverse weather conditions, which can significantly impact the model's performance.

While our current results are promising within laboratory settings, we acknowledge the necessity to validate the model's performance under real-world conditions. The transition from controlled environments to actual field applications is fraught with challenges, including variability in environmental conditions, hardware limitations, and the need for robust data collection mechanisms.

At present, we face constraints in resources that limit our ability to conduct extensive field tests. Despite these limitations, we are committed to exploring avenues that will allow us to integrate the WSA model into practical applications. Future work will involve collaborations with industry partners to access real-world data and facilitate on-site testing.

In conclusion, the WSA network model presents a substantial step forward in the field of foreign object detection on transmission lines. The model's enhanced performance and potential for practical applicability are evident. However, there is a clear path for future enhancements. By addressing the current limitations and exploring new research directions, we are confident that we can develop an even more accurate, efficient, and robust detection system. This will not only be a valuable asset in laboratory conditions but also a practical solution for real-world applications.

## Data availability statement

The original contributions presented in the study are included in the article/supplementary material, further inquiries can be directed to the corresponding author.

## Author contributions

YW: Conceptualization, Funding acquisition, Methodology, Project administration, Supervision, Visualization, Writing – original draft, Writing – review & editing. HT: Conceptualization, Methodology, Software, Validation, Writing – original draft, Writing – review & editing. TY: Formal analysis, Software, Validation, Writing – original draft, Writing – review & editing. ZS: Investigation, Visualization, Writing – review & editing. AH: Validation, Writing – original draft. HZ: Formal analysis, Writing – review & editing. SG: Data curation, Resources, Writing – review & editing. LZ: Data curation, Resources, Writing – review & editing.

## References

[B1] Chen J. Ma B. Ji C. Feng Q. Zhang J. Liu X.. (2023). Apple inflorescence recognition of phenology stage in complex background based on improved YOLOv7. Comput. Electr. Agric. 211:108048. 10.1016/j.compag.2023.108048

[B2] Chen J. Mai H. Luo L. Chen X. Wu K. (2021). “Effective feature fusion network in BIFPN for small object detection,” in 2021 IEEE international conference on image processing (ICIP), 699–703.

[B3] Cheng G. Lai P. Gao D. Han J. (2023). Class attention network for image recognition. Sci. China Inf. Sci. 66:132105. 10.1007/s11432-021-3493-7

[B4] Du X. Wan W. Sun C. Li C. (2023). “Weak-shot object detection through mutual knowledge transfer,” in Proceedings of the IEEE/CVF Conference on Computer Vision and Pattern Recognition, 19671–19680.

[B5] Guo M. H. Lu C. Z. Liu Z. N. Cheng M. M. Hu S. M. (2023). Visual attention network. Comput. Visual Media 9, 733–752. 10.1007/s41095-023-0364-2

[B6] Guo X. Liu Q. Sharma R. P. Chen Q. Ye Q. Tang S. Fu L. (2021). Tree recognition on the plantation using UAV images with ultrahigh spatial resolution in a complex environment. Remote Sensing 13:4122. 10.3390/rs13204122

[B7] Hanning N. M. Fernández A. Carrasco M. (2023). Dissociable roles of human frontal eye fields and early visual cortex in presaccadic attention. Nat. Commun. 14, 53–81. 10.1038/s41467-023-40678-z 37666805 PMC10477327

[B8] Hanzl A. Casement R. Imrichova H. Hughes S. J. Barone E. Testa A.. (2023). Functional E3 ligase hotspots and resistance mechanisms to small-molecule degraders. Nat. Chem. Biol. 19, 323–333. 10.1038/s41589-022-01177-2 36329119 PMC7614256

[B9] Hu K. Chen Z. Kang H. Tang Y. (2024a). 3D vision technologies for a self-developed structural external crack damage recognition robot. Autom. Constr. 159:105262. 10.1016/j.autcon.2023.105262

[B10] Hu K. Li Y. Zhang S. Wu J. Gong S. Weng L.. (2024b). FedMMD: a federated weighting algorithm considering non-IID and local model deviation. Exp. Syst. Appl. 237:121463. 10.1016/j.eswa.2023.121463

[B11] Islam M. M. Nooruddin S. Karray F. Muhammad G. (2023). Multi-level feature fusion for multimodal human activity recognition in internet of healthcare things. Inf. Fusion 94, 17–31. 10.1016/j.inffus.2023.01.015

[B12] Ju A. Wang Z. A. (2023). novel fully convolutional network based on marker-controlled watershed segmentation algorithm for industrial soot robot target segmentation. Evol. Int. 16, 963–980. 10.1007/s12065-022-00708-z

[B13] Koh M. S. (2023). “An end-to-end trainable power line communication system,” in Proceedings of the 2023 8th International Conference on Machine Learning Technologies, 257–263.

[B14] Kou R. Wang C. Peng Z. Zhao Z. Chen Y. Han J.. (2023). Infrared small target segmentation networks: a survey. Pattern Recognit. 143:109788. 10.1016/j.patcog.2023.109788

[B15] Li F. Zhang H. Xu H. Liu S. Zhang L. Ni L. M. Shum H. Y. (2023). “Mask dino: Towards a unified transformer-based framework for object detection and segmentation,” in Proceedings of the IEEE/CVF Conference on Computer Vision and Pattern Recognition, 3041–3050. 10.1109/CVPR52729.2023.00297

[B16] Li G. Hua Q. Sun L. Khosravi. A Pabon J. (2023). Thermodynamic modeling and optimization of hybrid linear concentrating photovoltaic and mechanically pumped two-phase loop system. Applied Energy 333:120547. 10.1016/j.apenergy.2022.120547

[B17] Li K. Wang Y. Zhang J. Gao P. Song G. Liu Y.. (2023). “Uniformer: unifying convolution and self-attention for visual recognition,” in IEEE Transactions on Pattern Analysis and Machine Intelligence. 10.1109/TPAMI.2023.3282631 37276098

[B18] Li Y. Hou Q. Zheng Z. Cheng M. M. Yang J. Li X. (2023). “Large selective kernel network for remote sensing object detection,” in Proceedings of the IEEE/CVF International Conference on Computer Vision (16794–16805). 10.1109/ICCV51070.2023.01540

[B19] Liang D. Wang S. Chen N. Chu B. Li Y. Tao H.. (2023). Research on foreign body identification method of transmission line based on improved Yolov5s. Third international conference on machine learning and computer application (ICMLCA 2022). SPIE 12636, 352–355. 10.1117/12.2675367

[B20] Liang H. Zuo C. Wei W. (2020). Detection and evaluation method of transmission line defects based on deep learning. IEEE Access 8, 38448–38458. 10.1109/ACCESS.2020.2974798

[B21] Lin J. Yang C. K. Kao Y. F. (2024). Three-dimensional positioning on image point of interest via google geographic information. Signal Image Video Proc. 22, 1–9. 10.1007/s11760-023-02947-8

[B22] Liu H. Li Z. Hall D. Liang P. Ma T. (2023). Sophia: A scalable stochastic second-order optimizer for language model pre-training. arXiv Preprint arxiv:2305.14342. 10.48550/arXiv.2305.14342

[B23] Liu L. Cai L. Zhang C. Zhao X. Gao J. Wang W. (2023). “Linrec: Linear attention mechanism for long-term sequential recommender systems,” in Proceedings of the 46th International ACM SIGIR Conference on Research and Development in Information Retrieval, 289–299.

[B24] Liu Q. Ye H. Wang S. Xu Z. (2024). YOLOv8-CB: dense pedestrian detection algorithm based on in-vehicle camera. Electronics 13, 236. 10.3390/electronics13010236

[B25] Liu X. Peng H. Zheng N. Yang Y. Hu H. Yuan Y. (2023). “Efficientvit: Memory efficient vision transformer with cascaded group attention,” in Proceedings of the IEEE/CVF Conference on Computer Vision and Pattern Recognition, 14420–14430.

[B26] Lue N. Z. Garcia E. M. Ngan K. C. Lee C. Doench J. G. Liau B. B. (2023). Base editor scanning charts the DNMT3A activity landscape. Nat. Chem. Biol. 19, 176–186. 10.1038/s41589-022-01167-4 36266353 PMC10518564

[B27] Luo Y. Yu X. Yang D. Zhou B. (2023). A survey of intelligent transmission line inspection based on unmanned aerial vehicle. Artif. Int. Rev. 56, 173–201. 10.1007/s10462-022-10189-2

[B28] Pan Y. Ao X. Hu K. Kang H. Jin Y. Chen Y.. (2024). ODN-Pro: an improved model based on YOLOv8 for enhanced instance detection in orchard point clouds. Agronomy 14:697. 10.3390/agronomy14040697

[B29] Sun Y. Tang H. Zhang H. (2024). Automatic detection of pavement marking defects in road inspection images using deep learning. J. Perf. Constr. Fac. 38:04024002. 10.1061/JPCFEV.CFENG-4619

[B30] Talaat F. M. ZainEldin H. (2023). An improved fire detection approach based on YOLO-v8 for smart cities. Neur. Computi. Appl. 35, 20939–20954. 10.1007/s00521-023-08809-1

[B31] Vahdani E. Tian Y. (2024). Overview of temporal action detection based on deep learning. Artif. Int. Rev. 57:26. 10.1007/s10462-023-10650-w35877805

[B32] Vidit V. Engilberge M. Salzmann M. (2023). “Clip the gap: A single domain generalization approach for object detection,” in Proceedings of the IEEE/CVF Conference on Computer Vision and Pattern Recognition, 3219–3229. 10.1109/CVPR52729.2023.00314

[B33] Wang B. Wu R. Zheng Z. Guo Z. (2017). “Study on the method of transmission line foreign body detection based on deep learning,” in 2017 IEEE Conference on Energy Internet and Energy System Integration (EI2). IEEE, 1–5.

[B34] Wang K. Liew J. H. Zou Y. Zhou D. Feng J. (2019). “Panet: Few-shot image semantic segmentation with prototype alignment,” in Proceedings of the IEEE/CVF International Conference on Computer Vision, 9197–9206.

[B35] Wang Q. Si G. Qu K. Gong J. Cui L. (2022). Transmission line foreign body fault detection using multi-feature fusion based on modified YOLOv5. J. Phys. Conf. Series IOP Publishing 2320, 12–28. 10.1088/1742-6596/2320/1/012028

[B36] Wang Z. Li Y. Chen X. Lim S. N. Torralba A. Zhao H. Wang S. (2023a). “Detecting everything in the open world: towards universal object detection,” in Proceedings of the IEEE/CVF Conference on Computer Vision and Pattern Recognition, 11433–11443. 10.1109/CVPR52729.2023.01100

[B37] Wang Z. Yuan G. Zhou H. Ma Y. Ma Y. (2023b). Foreign-object detection in high-voltage transmission line based on improved YOLOv8m. Appl. Sci. 13:12775. 10.3390/app132312775

[B38] Wu Q. Li X. Wang K. Bilal Hazrat. (2023). Regional feature fusion for on-road detection of objects using camera and 3D-LiDAR in high-speed autonomous vehicles. Soft Comput. 27, 18195–18213. 10.1007/s00500-023-09278-3

[B39] Zhang T. Li L. Cao S. Pu T. Peng Z. (2023). “Attention-guided pyramid context networks for detecting infrared small target under complex background,” in IEEE Transactions on Aerospace and Electronic Systems. 10.48550/arXiv.2111.03580

[B40] Zhang X. P. Yang X. M. Xie W. Q. Liu Q. S. Tang S. H. (2023). Comparison and selection of index for macro-indentation test of brittle rock. Rock Mech. Rock Eng. 56, 6375–6394.

[B41] Zheng D. Dong W. Hu H. Chen X. Wang Y. (2023). “Less is more: focus attention for efficient detr,” in Proceedings of the IEEE/CVF International Conference on Computer Vision, 6674–6683. 10.1109/ICCV51070.2023.00614

[B42] Zhu L. Wang X. Ke Z. Zhang W. Lau R. W. (2023). “Biformer: Vision transformer with bi-level routing attention,” in Proceedings of the IEEE/CVF Conference on Computer Vision and Pattern Recognition, 10323–10333.38379942

